# Administración local de prostaglandinas para acelerar el movimiento ortodóntico. Revisión de la literatura

**DOI:** 10.21142/2523-2754-1203-2024-211

**Published:** 2024-09-17

**Authors:** Alejandro Martínez-Aldave, Gissella Gutiérrez Tapia

**Affiliations:** 1 División de Ortodoncia, Universidad Científica del Sur. Lima, Perú. 100005702@cientifica.edu.pe , ggutierrezt@cientifica.edu.pe Universidad Científica del Sur División de Ortodoncia Universidad Científica del Sur Lima Peru 100005702@cientifica.edu.pe ggutierrezt@cientifica.edu.pe

**Keywords:** prostaglandinas, movimiento dental acelerado, administración local, prostaglandins, accelerated tooth movement, local administration

## Abstract

**Objetivo:**

La búsqueda de optimizar el movimiento dentario y reducir el tiempo de tratamiento es de gran interés para el ortodoncista, por lo cual se innova con nuevos materiales y procedimientos que permitan alcanzar los objetivos de tratamiento en un corto plazo. La administración de prostaglandinas ha sido estudiada y se ha comprobado su efecto acelerador del movimiento dentario al ser administradas localmente. El objetivo de este estudio fue evaluar los últimos alcances sobre los efectos de la administración local de prostaglandinas para acelerar el movimiento dentario.

**Metodología:**

Se realizó una búsqueda en cuatro bases de datos (PubMed, SciELO, Cochrane, Google Scholar) y tres revistas especializadas (AJODO, Angle Orthodontics, Journal of Clinical Orthodontics) hasta agosto de 2023. La calidad metodológica de los estudios fue analizada usando la lista de verificación PRISMA.

**Resultados:** El estudio contó con 14 artículos. La administración local de prostaglandinas acelera la velocidad del movimiento dentario e incrementa el número de osteoclastos. Se ha reportado evidencia de reabsorción radicular en relación con altas dosis de prostaglandinas por la vía submucosal, y una reducción de la reabsorción radicular administrando prostaglandinas con gluconato de calcio. La administración local de prostaglandinas por la vía submucosal produce un dolor de leve a moderado, y se recomienda su inoculación junto a un anestésico local; sin embargo, la administración oral del compuesto análogo Misoprostol no produce dolor ni evidencia de reabsorción radicular.

**Conclusiones:**

No se ha encontrado evidencia científica suficiente que sustente la administración local de prostaglandinas como un método seguro para acelerar el movimiento dentario en humanos.

## Abstract (en)

**Objective:**

The search to optimize tooth movement and reduce treatment time is of great interest to the orthodontist, as it involves innovating new materials and procedures to achieve the treatment objectives in the short term. The administration of prostaglandins has been studied, and their effect on accelerating tooth movement when administered locally has been proven. The objective of this study was to evaluate the latest achievements concerning the effects of local administration of prostaglandins to accelerate tooth movement for orthodontic purposes.

**Methodology:**

A search was conducted in four databases (PubMed, SciELO, Cochrane, Google Scholar) and three specialized journals (AJODO, Angle Orthodontics, Journal of Clinical Orthodontics) until August 2023. The methodological quality of the studies was analyzed using the PRISMA checklist.

**Results:**

The study had 14 articles. Local administration of prostaglandins accelerates the speed of tooth movement and increases the number of osteoclasts. Evidence of root resorption has been reported concerning high doses of prostaglandins via the submucosal route and a reduction in root resorption by administering prostaglandins with calcium gluconate. Local administration of prostaglandins via the submucosal route produces mild to moderate pain, and inoculation with a local anesthetic is recommended. However, oral administration of the analogous compound Misoprostol does not produce pain or evidence of root resorption.

**Conclusions:**

Sufficient scientific evidence has not been found to support the local administration of prostaglandins as a safe method to accelerate tooth movement in humans.

## INTRODUCCIÓN

Acelerar el movimiento dentario y reducir el tiempo de tratamiento ortodóntico son temas de investigación constante debido a su importancia clínica e interés compartido, tanto por el paciente y como por el ortodoncista ^1^^-^^4^. Los procedimientos menos invasivos son los mejor aceptados por los pacientes ^5^, como el uso de láser de baja frecuencia, dispositivos vibrátiles y administración de medicamentos, entre los cuales se encuentran las prostaglandinas (PG) ^6^.

Las prostaglandinas se encuentran naturalmente en el cuerpo y participan en varias funciones fisiológicas, entre las cuales está la de ser un mediador de la inflamación, por lo que, además, se reporta su influencia en el movimiento dentario. Las prostaglandinas E2 (PGE2) son producidas principalmente por los fibroblastos del ligamento periodontal y los osteoblastos ^7^, y entre sus funciones están las de regular la producción de cAMP (adenosina monofosfato cíclica), un mediador de la diferenciación celular, el cual es precursor de la aparición de los osteoclastos en el periodonto del diente durante el movimiento ortodóntico ^6^^,^^8^^-^^10^. Estudios realizados en animales por Lee, Leiker y Boeknoogen han reportado que la administración local de prostaglandinas en diferentes concentraciones acelera el movimiento dentario, genera dolor de leve a moderado al ser inyectada localmente, y se ha evidenciado reabsorción radicular en altas dosis ^11^. Los estudios en humanos de Yamasaki y Patil reportaron que la administración de prostaglandinas genera un movimiento dentario de 1,6 a 3 veces más rápido que el movimiento dentario en el grupo control sin efectos secundarios evidentes ^4^.

Los estudios en humanos han demostrado la efectividad de las prostaglandinas al ser aplicadas localmente, lo que incrementa la velocidad del movimiento dentario y no se han encontrado alteraciones periodontales ni patologías posteriores a la aplicación infiltrativa ^13^. Sin embargo, la limitada experimentación en humanos podría deberse al riesgo de reabsorción radicular que sigue a la administración de prostaglandinas por la vía submucosal, por lo que esta revisión también recopiló las experimentaciones en animales, para determinar cuáles formulaciones de prostaglandinas solas, y en combinación con otros compuestos, podrían presentar efectos benéficos. Por ello, el presente estudio tuvo como propósito revisar la información actualizada respecto del uso local de prostaglandinas para acelerar el movimiento dentario en humanos, y hacer una revisión de las experimentaciones en animales y sus resultados.

## MATERIALES Y MÉTODOS

Esta revisión de la literatura fue registrada en la Universidad Científica del Sur con el número Nº POS-95-2023-00493.

### Estrategia de búsqueda

La búsqueda del material bibliográfico fue ejecutada hasta el 13 de agosto de 2023, en 4 bases de datos: PubMed, SciELO, Google Scholars y Cochrane, utilizando las palabras clave “prostaglandina”, “movimiento dental acelerado”, “administración local”. Adicionalmente, se buscó manualmente en las revistas acreditadas *American Journal of Orthodontics and Dentofacial Orthopedics* (AJODO), *Angle Orthodontist y Journal of Clinical Orthodontics* (JCO). Los criterios de búsqueda se describen en la Tabla 1.

**Tabla 1:** Estrategia de búsqueda de descriptores de las diferentes bases de datos

Fuente	Fecha de búsqueda	Keywords	Resultados
Medline/PubMed	13/08/2023	“prostaglandin” AND “local administration” AND “accelerated tooth movement”). Filters: Publication year from 2018 to 2023.	1
SciELO	13/08/2023	“prostaglandin” AND “local administration” AND “accelerated tooth movement”). Filters: Publication year from 2018 to 2023.	1
Cochrane	13/08/2023	“prostaglandin” AND “local administration” AND “accelerated tooth movement”). Filters: Publication year from 2018 to 2023.	0
Google Scholars	13/08/2023	“prostaglandin” AND “local administration” AND “accelerated tooth movement”). Filters: Publication year from 2018 to 2023.	41
*AJODO*	13/08/2023	“prostaglandin” AND “local administration” AND “accelerated tooth movement”). Filters: Publication year from 2018 to 2023.	14
*Angle Orthodontist*	13/08/2023	“prostaglandin” AND “local administration” AND “accelerated tooth movement”). Filters: Publication year from 2018 to 2023.	5
*Journal of Clinical Orthodontics*	13/ 08/2023	“prostaglandin” AND “local administration” AND “accelerated tooth movement”). Filters: Publication year from 2018 to 2023.	0

### Proceso de calibración

El proceso de calibración fue realizado por el investigador principal (AJMA) y el segundo autor (GGT). Se seleccionaron 10 estudios y se obtuvo un 100% de concordancia en la búsqueda de artículos y extracción de los resultados de interés.

### Recolección de datos

**Figura 1:**
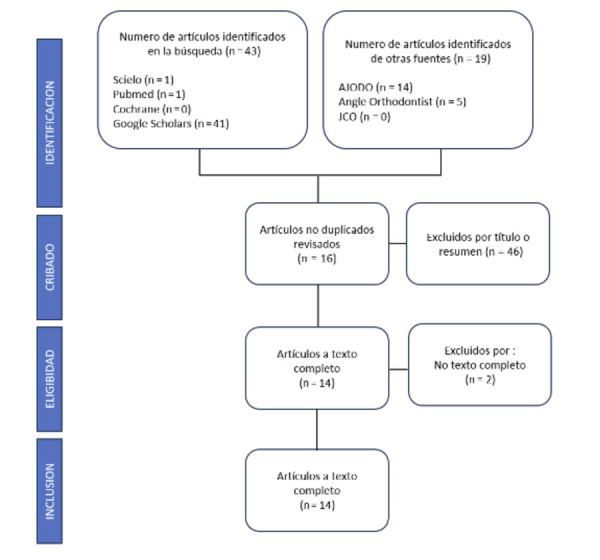
Diagrama de flujo PRISMA de la revisión de la literatura

Los estudios recolectados fueron analizados según el título y resumen. El investigador principal revisó los artículos y su relevancia para esta investigación. En caso de dudas, estas fueron resueltas por el segundo autor. El proceso se describe en la Figura 1.

### Manejo de datos

El investigador sintetizó los datos de los estudios relacionados con el tema de investigación en la Tabla 2, indicando el autor, año, país, antecedentes, resultados y conclusiones. Fueron 14 artículos tomados en cuenta para esta investigación. Los estudios experimentales realizados en animales entre 1980 y 2018 se incluyeron en la Tabla 3.

**Tabla 2:** Síntesis de los estudios seleccionados para la revisión.

N.°	Autor, año, país	Diseño de estudio	Antecedentes relacionados	Variables	Resultados	Conclusión
1	Patil A, *et al*. (2018). Reino Unido	Revisión de literatura	Yamasaki (1984), Leiker (1995), Kale (2004)	PGE_1_, PGE_2_	Cantidades mínimas de PGE_1_ causan incremento en el movimiento dentario e hiperalgesia. El misoprostol (análogo PGE_1_) es efectivo para incrementar el movimiento ortodóntico.	Hay incremento en la demanda de reducir el tiempo de tratamiento. El ortodoncista debe escoger de acuerdo con la necesidad el caso.
2	Unnam D, *et al*. (2018). India	Revisión de literatura	Yamasaki (1980, 1984), Seifi (2003)	PGE_1_, PGE_2_ + calcio	Hay incremento del número de osteoclastos. La administración de PG puede acelerar el movimiento dentario.	El uso combinado de PGE_2_ y calcio puede incrementar significativamente el movimiento dentario.
3	Ciur MD, *et al*. (2019). Rumanía	Revisión de literatura	Yamasaki (1982), Leiker (1995), Seifi (2003)	PGE_1_, PGE_2_ + calcio	Hay incremento del movimiento dentario comparado con grupo control luego de aplicación única o múltiple.	El mecanismo de acción de PGE_2_: activación directa de osteoclastos, tiene como resultado reabsorción ósea.
4	Eltimamy A, *et al*. (2019). Egipto	Revisión sistemática	Sekhavat (2002), Lee (1990), Yamasaki (1984), Spielmann (1989)	PGE_1_, PGE_2_	La prostaglandina mostró un incremento marcado del movimiento dentario. No se encontró evidencia de efectos secundarios en la gingiva y hueso tras la inoculación.	Hay evidencia inconclusa sobre la aceleración del movimiento dentario.
5	Kaur A, *et al*. (2019). India	Revisión de literatura	Yamasaki (1980, 1982, 1984), Leiker (1995), Seifi (2003)	PGE_1_, PGE_2_	Hay evidente relación entre PG, aplicación de fuerza y aceleración del movimiento dentario. La aceleración del movimiento dentario está en relación con el número y la dosis administrada.	Los estudios en humanos demuestran que la administración de PGE_2_ acelera la retracción de caninos en 1,6 veces frente al grupo control. La PG con Ca estabiliza la reabsorción radicular.
6	Kulshrestha R, *et al*. (2019). India	Revisión de literatura	Yamasaki (1984)	PGE_1_	Las prostaglandinas incrementan el número de osteoclastos, lo que causa reabsorción ósea.	La administración local de PG podría ser un método seguro y efectivo para el movimiento dentario.
7	Talla R, *et al*. (2020). India	Revisión de literatura	Seifi (2003), Sehhavat (2002)	PGE_2_	La aplicación de PG incrementa la velocidad del movimiento dentario entre 1,5 a 3 veces, sin efectos significativos de reabsorción radicular.	Esta técnica de aceleración presenta algunos inconvenientes.
8	Abbas N, *et al.* (2021). Irak	Revisión de literatura	Seifi (2003)	PGE_2_ + calcio	La PG estimula la reabsorción ósea al actuar directamente sobre los osteoclastos, lo que acelera el movimiento dentario.	La PG en diferentes concentraciones puede causar reabsorción radicular. La PG con Ca estabiliza la reabsorción radicular.
9	Aruq SA, *et al*. (2021). EE. UU.	Revisión sistemática	Yamasaki (1980, 1982, 1984), Kale (2004), Spielmann (1989), Patil (2005)	PGE_1_, PGE_2_	La PG acelera el movimiento dentario y eleva el nivel de las metaloproteinasas. La tasa de movimiento dentario es de 1-2 mm por mes.	Hay evidencia no concluyente debido a la muestra pequeña y el corto tiempo.
10	Krishnan V, *et al*. (2021). EE. UU.	Revisión de literatura	Yamasaki (1980, 1982, 1984), Leiker (1995), Patil (2005), Seifi (2003)	PGE_1_, PGE_2_ + calcio	Una dosis de PG (1 ug) produce un aumento significativo del movimiento dentario, con una tasa de 1,7 a 1. La PG con Ca no mostró una reducción estadísticamente significativa de la reabsorción radicular, pero sí una leve disminución en el movimiento dentario frente a la aplicación sola de prostaglandinas.	Existe evidencia inconclusa concerniente a la aceleración del movimiento dentario. Se requiere más investigación.
11	Virdi G, *et al*. (2021). India	Revisión de literatura	Yamasaki (1980, 1982)	PGE_1_	La PG incrementa el número de osteoclastos y la cantidad de reabsorción ósea.	Hay limitada evidencia en humanos. Aún está en fase experimental.
12	Alfailany DT, *et al*. (2021). Siria	Revisión sistemática	Rajasekaran (2014), Patil (2005)	PGE_2_	La PG puede causar dolor y reabsorción radicular según la dosis.	La calidad de la evidencia es baja. Las investigaciones futuras deben evaluar posibles efectos secundarios.
13	Gupta S, *et al*. (2022). India	Revisión de literatura	Lee (1990), Spielmann (1989), Seifi (2003)	PGE_1_, PGE_2_ + calcio	La PG estimula la reabsorción ósea al activar los osteoclastos. La administración semanal de PG cambia la velocidad del movimiento dentario sin lesiones aparentes. La PG produce dolor al ser inoculada.	Es posible incrementar el anclaje biológicamente en sitios específicos al suprimir las prostaglandinas.
14	Lin Y, *et al*. (2022). EE. UU.	Revisión de literatura	Cağlaroğlu (2012), Cui (2018), Leiker (1995), Seifi (2003, 2015)	PGE_2_, PGE_2_ + akebiasaponin, PGE_2_ + calcio, PGE_2_ + TH	Diferentes dosis de PGE_2_ muestran un incremento del movimiento dentario, así como reabsorción radicular. La PGE_2_ con hormona tiroidea acelera levemente el movimiento dentario. La PGE_2_ con hormona tiroidea acelera la tasa de movimiento dentario de moderado a alto. La combinación de PG con HT o Ca reduce la reabsorción radicular.	La PG con Ca reduce la cantidad de reabsorción radicular. Se requieren más estudios.

**Tabla 3:** Experimentos en animales para acelerar el movimiento dentario

ID	Autor, país, año	Tipo y tamaño de muestra	Vía de administración y biomaterial	Dosis	Aparatología y/o fuerza	Periodo	Resultados
1	Yamasaki, Japón, 1980	35 ratas	Submucosal PGE_1_ y PGE_2_	0,1 *u*g, 1 *u*g, 10 *u*g	1 separador de 0,25 mm entre las molares	3 días	PGE_1_ y PGE_2_ aumentaron el número de osteoclastos: dosis 1 ug y 10 ug. Dosis 1 ug, PGE_2_ aumentó del número de osteoclastos (16,5 ± 1,9) vs. PGE_1_ (7,7 ± 1,9). Dosis 10 ug: PGE_1_ y PGE_2_ tuvieron similar número de osteoclastos (20,4 y 21,4, respectivamente).
2	Yamasaki, Japón, 1982	2 monos	Submucosal PGE_1_ y PGE_2_	40 *u*g	Resorte abierto 100 g. ATP, arco redondo cinchado	18 días/4 semanas	Retracción de caninos (extracciones primeras premolares) Experimento 1. 6 dosis de PGE_2_ (día 0, 1, 5, 9, 12, 15) Día 18: movimiento dentario PGE_2_ (1,3 mm) vs. control (0,5 mm). Experimento 2. Semanas1 y 2 (PG lado izquierdo, control lado derecho), Semanas 3 y 4 (PG lado derecho, control lado izquierdo). PG presentó un movimiento dentario x 2 vs. lado control durante las semanas 1 y 2. Fin de semana 4: no hubo diferencias significativas entre ambos grupos.
3	Kang, Corea del Sur, 1983	30 ratas	Submucosal PGE_2_	0,2 *u*g, 0,4 *u*g, 0,8 *u*g, 1 *u*g	Separador clamp (0,014)	3 días	PGE_2_ aumentó el número de osteoclastos vs. lado control. Dosis 0,8 ug y 1 ug mostraron un aumento significativo de osteoclastos vs. control.
4	Chao, China, 1988	20 ratas	Submucosal PGE_2_	50 *u*g	1 separador 1,2 mm entre las molares	5 días	Experimento: Dosis diarias 50 ug. PGE_2_ presentó un descenso inmediato en el número de fibroblastos y un aumento en el número de osteoclastos. Día 5: PGE_2_ presentó un descenso en el número de fibroblastos y osteoclastos.
5	Lee, China, 1989	25 gatos	Submucosal PGE_2_	10 *u*g	Resorte de NiTi cerrado, 80 g	14 días	Día 1: PGE_2_ presentó un movimiento dentario mayor vs. lado control. Día 7 al día 10: Lado PGE_2_ presentó un mínimo movimiento dentario. Día 4 al día 10: Lado PGE_2_ presentó una reducción en zonas de reabsorción. Día 14: Movimiento dentario PGE_2_ (2,14 mm) vs. control (1,39 mm).
6	Lee, China, 1990	72 ratas	Administración local vs. sistémica PGE_1_	L: 5 *u*g S: 7,5 ng/kg	1 separador 0,5 mm entre las molares	5 días	Administración sistémica tuvo un efecto mayor vs. administración local (aumento en el número de osteoclastos y lagunas de reabsorción). Día 5: se evidenció un aumento de la actividad de reabsorción en todos los grupos. PGE_2_ presentó un mayor número de osteoclastos y lagunas de reabsorción vs. control.
7	Lee, China, 1990	160 ratas	Submucosal PGE_2_ vs. aceite de semilla de onagra	PGE_2_ (5 *u*g) Onagra (10 mg)	1 elástico 3/16” (50 g)	7 días	Día 3: Lado PG presentó un pico en el número de osteoclastos, seguido por el grupo de aceite de semilla de onagra y último el grupo control. Días 3 y 5: Lado PG presentó fibroblastos fagocitando fragmentos colágenos. Día 7: Lado PG evidenció fibroblastos formando nuevas fibras colágenas. Lado PG presentó una mayor reabsorción radicular vs. lado control.
8	Brudvik, Noruega, 1991	25 ratas	Submucosal PGE_2_	10 *u*g	Resorte NiTi cerrado, 50 g	10 días	Día 3. PGE_2_ con dosis única no presentó signos de reabsorción radicular. Día 7. PGE_2_ con 3 dosis, se evidencia mayor reabsorción radicular vs. lado control. Día 10. PGE_2_ con 4 dosis, se presentó el doble de la reabsorción radicular vs. lado control.
9	Leiker, EE. UU., 1995	132 ratas	Submucosal PGE_2_	0 *u*g, 1 *u*g, 5 *u*g, 10 *u*g	Resorte NiTi cerrado, 60 g	4 semanas	Dosis única presentó los mismos resultados que múltiples dosis. PGE_2_ presentó una mayor reabsorción radicular vs. lado control. Dosis 0,1 *u*g es suficiente para comenzar movimiento dentario. Dosis más altas están asociadas con mayor reabsorción radicular.
10	Boekenoogen, EE. UU., 1996	132 ratas	Submucosal PGE_2_	0,1 *u*g, 1 *u*g, 5 *u*g, 10 *u*g	Resorte NiTi cerrado, 60 g	4 semanas	Dosis 0,1 *u*g. No presentó reabsorción radicular. Dosis única 10 *u*g. Presentó un aumento de la reabsorción radicular. Dosis semanales de 10 *u*g, presentaron mayor reabsorción radicular vs. dosis 0,1 *u*g.
11	Sekhavat, Irán, 2002	64 ratas	Oral (sonda gástrica). Misoprostol (PGE_1_ análogo)	2,5 *u*g, 5 ug, 10 *u*g, 25 *u*g, 50 ug, 100 ug	Resorte NiTi cerrado, 60 g	2 semanas	Misoprostol no generó reabsorción radicular. Dosis 10 *u*g o 25 ug presentaron efectos similares a dosis 50 ug o 100 *u*g. Misoprostol. 25 ug dosis ideal para acelerar el movimiento dentario con mínima reabsorción radicular. Todas las dosis no presentaron reabsorción radicular significativa.
12	Seifi, Irán, 2003	24 ratas	Submucosal PGE_2_ + gluconato de Ca	PG (100 *u*g), Ca 10% (200 mg/kg)	Resorte NiTi cerrado, 60 g	21 días	Movimiento dentario: PGE_2_ (0,47 mm), PGE_2_ + Ca (0,40 mm), control (0,21 mm). PGE + Ca y control: reabsorción radicular no fue significativa. PGE_2_ + Ca: mayor movimiento dentario y menor reabsorción radicular vs. lado control.
13	Kale, Turquía, 2004	37 ratas	Submucosal PGE_2_ vs Vitamina D	PG (0.1*u*g) Vitamina D 20 uL 10-10 MOL/L	*Loop* (0,012 SS). 20 g	9 días	Movimiento dentario: PGE_2_ (2,16 mm), vitamina D (2,11 mm), control (1,72 mm). Numero de osteoblastos: vitamina D (25,1/mm^2^) vs PGE_2_ (20,8/mm^2^). PGE_2_ presentó una mayor cantidad de capilares sanguíneos vs. lado vitamina D.
14	Cağlaroğlu, Turquía, 2012	45 conejos	Submucosal PGE_2_	10*u*g	Resorte (0,011 SS). 20 g	14 días	Vía submucosal. Incremento en el número de fibroblastos, neovascularización y osteoclastos. Orden de acuerdo con grado de respuesta: intraligamental > submucosal > intravenosa > control. Vía intraligamental presentó la mayor cantidad de células (osteoblastos y osteoclastos).
15	Seifi, Irán, 2015	64 ratas	Submucosal PGE_2_+gluconato Ca+Hormona Tiroidea	PG: 100*u*g Ca: 10% (200 mg/kg) TH: 20 *u*g	Resorte NiTi cerrado, 60 g	21 días	PGE_2_ + TH. Aumento del movimiento dentario (0,73 mm) vs. control (0,23 mm). Movimiento dentario: PGE_2_ + Ca + TH (0,65 mm) vs. PGE_2_ (0,4) Movimiento dentario: TH (0,45 mm), Ca (0,2 mm) y Ca + TH (0,36) Reabsorción radicular: No se encontró diferencias entre los grupos y control.
16	Mohamed, Egipto, 2018	10 conejos	PGE_2_ + Vitamina D	PG: 100*u*g VitD: 1.5 IU/mL	Resorte NiTi cerrado, 60 g	21 días	Movimiento dentario: PGE_2_ + VitD (1,43 mm), control (0,66 mm). No se evidenció irritación en los tejidos blandos.
17	Cui, China, 2018	40 ratas	PGE_2_ vs Akebia Saponina D	PG2: 25 *u*g ASD1: 5mg/Kg ASD2: 10mg/Kg	Resorte NiTi cerrado, 40 g	28 días	Movimiento dentario mayor en PGE_2_, ASD1 y ASD2 vs. control. Día 3. PGE_2_ presentó mayor movimiento dentario significativo vs. lado control. Día 7. PGE_2_ y ASD2 presentaron mayor movimiento dentario significativo vs. lado control. PGE_2_ y ASD2 presentaron efectos similares, sin diferencias significativas.

## RESULTADOS

### Efectos de las prostaglandinas en el movimiento dentario 

Cinco artículos atribuyeron el aumento en la velocidad del movimiento dentario a un incremento en el número de osteoclastos y un aumento de la reabsorción ósea en la zona de compresión. La cantidad del aumento de la velocidad del movimiento dentario vinculado a la aplicación de prostaglandinas frente al lado control varió dependiendo del compuesto utilizado (PG1 o PG2), el tipo de aparatología (resorte de níquel titanio, separadores elásticos, cadenas de poder), y el uso o no de compuestos químicos que puedan potenciar los efectos de las prostaglandinas (gluconato de calcio, vitamina D, hormona tiroidea).

La experimentación con prostaglandinas con fines ortodónticos se remonta a los estudios de Yamasaki *et al*. ^13^^,^^14^, quienes, por medio de la inoculación local (submucosal) de prostaglandinas en ratas y monos, encontraron que ambas PGE1 y PGE2 aumentaban la cantidad de osteoclastos, y que la velocidad del movimiento dentario era cerca de 2 veces más rápida que en el lado control. A su vez, encontró que la administración o supresión de las prostaglandinas, potenciaba y desaceleraba, respectivamente, el movimiento dentario en curso ^15^^-^^17^.

Estudios subsecuentes experimentaron en ratones, traccionando los incisivos superiores en sentido posterior con resortes de níquel titanio cerrados (40-80 g) y anclaje en los molares. Lee *et al*. ^18^ y Cui *et al*. ^19^ demostraron que la administración de PGE2 generaba un movimiento dentario mayor al del lado control. El aumento en el número de osteoclastos y lagunas de reabsorción generó un movimiento dentario acelerado desde el día de la aplicación. Lee *et al*. ^20^ comparó los efectos de la administración local y la administración sistémica de prostaglandinas en ratas, y encontró efectos mayores en la administración sistémica, con mayor número de osteoclastos y lagunas de reabsorción.

Cağlaroğlu *et al*. ^21^ compararon las vías de administración de PGE2: intravenosa, submucosal e intraligamental. La vía submucosal presentó un incremento del número de fibroblastos y neovascularización; sin embargo, fue la vía intraligamental la que presentó el mayor aumento de células (osteoblastos y osteoclastos) y efectos más intensos.

Otros estudios han combinado las prostaglandinas con diferentes compuestos, para evaluar su sinergismo o antagonismo. Yamasaki *et al*. ^13^ y Lee *et al*. ^20^ reportaron que los antiinflamatorios, en especial la indometacina (inhibidor selectivo de las prostaglandinas), reducían la velocidad del movimiento dentario y el número de osteoclastos. Por el contrario, el uso combinado de prostaglandinas con gluconato de calcio, hormona tiroidea o vitamina D, han demostrado tener un efecto potenciador en el movimiento dentario. Seifi *et al*. ^22^ administraron PGE2 con gluconato de calcio y encontraron que la velocidad del desplazamiento dentario con la administración única de PGE2 era superior, seguido por la combinación PGE2 con gluconato de calcio y, por último, el grupo control. Sin embargo, la reabsorción radicular fue menor en el grupo PGE2 con gluconato de calcio.

Kale *et al*. ^23^ compararon las prostaglandinas y la vitamina D, y hallaron que ambas potenciaban el movimiento dentario. La PGE2 fue superior en la cantidad de osteoclastos y lagunas de reabsorción, mientras que la vitamina D fue superior en el conteo de osteoblastos. Mohamed *et al*. ^24^ combinaron la PGE2 con vitamina D y encontraron que el movimiento dentario era cercano al doble que el lado control, y no hallaron irritación en los tejidos blandos. Seifi *et al*. ^25^ combinaron prostaglandinas, gluconato de calcio y hormona tiroidea (TH), y encontraron que la combinación de PGE2 con hormona tiroidea obtuvo los niveles de movimiento dentario más altos, seguidos por la administración de PGE2 con gluconato de calcio y hormona tiroidea. No hallaron diferencias significativas en la reabsorción radicular entre los grupos experimentales y control.

### Reabsorción radicular en animales

Cinco artículos mencionan el riesgo de reabsorción radicular como un efecto de la aplicación local de prostaglandinas. Se ha reportado una relación entre la dosis administrada y la aparición de signos de reabsorción radicular. Sin embargo, la combinación de prostaglandinas con gluconato de calcio u hormona tiroidea ha demostrado reducir la cantidad de reabsorción radicular y, a su vez, acelerar el movimiento dentario, siendo la administración única de prostaglandinas el método más veloz, seguido por la combinación de prostaglandinas con gluconato de calcio u hormona tiroidea, y, finalmente, el grupo control.

Estudios han evaluado el potencial de reabsorción radicular de la administración de prostaglandinas. Brudvik y Rygh ^26^ experimentaron con la administración de PGE2. A los 3 días de una dosis única de 10 *u*g de PGE2 no se evidenciaron signos de reabsorción radicular. Luego de 10 días con 4 dosis de 10 *u*g se generó el doble de reabsorción radicular que el lado control. 

Leiker *et al*. ^27^ compararon diferentes dosis (0,1 *u*g, 1 *u*g, 5 *u*g y 10 *u*g) de PGE2 administradas de manera única y en dosis semanales, y encontraron que una dosis de 0,1 *u*g era suficiente para acelerar el movimiento dentario, y que dosis únicas tenían un efecto similar a administrar dosis repetidas. Reportó también que dosis más altas presentaban niveles de reabsorción radicular mayores. Boekenoogen *et al*. ^28^ repitieron los parámetros de Leiker y reportaron que una dosis única de 0,1 *u*g no generaba reabsorción radicular evidente, mientras que dosis de 10 *u*g semanales presentaban niveles de reabsorción radicular mayores. Sekhavat *et al*. ^29^ administraron misoprostol, un análogo de las prostaglandinas, por la vía sistémica, a un grupo de 64 ratas. Se traccionó los incisivos superiores en sentido posterior con un resorte que ejercía 60 g, mientras se administraba el compuesto por vía oral. Las dosis de 10 ug y 25 ug reportaron efectos similares a las de 50 *u*g y 100 *u*g, siendo la dosis de 25 *u*g la que obtuvo mejores valores respecto de la velocidad de movimiento dentario, con mínima evidencia de reabsorción radicular. Se concluyó que el misoprostol no generaba reabsorción radicular significativa ni efectos adversos como la hiperalgesia ^30^.

### Administración local de prostaglandinas en humanos

Con referencia a los ensayos clínicos en humanos, fueron 8 los artículos que mencionan los resultados del uso experimental de prostaglandinas en humanos, y reportan un movimiento dental acelerado, sin observar alteraciones en los tejidos de soporte. Sin embargo, 6 de estos artículos concluyen que la administración local de prostaglandinas aún no es un método seguro en humanos, y argumentan que se necesitan estudios a largo plazo y una muestra mayor.

Son 5 los estudios clínicos encontrados en la literatura que han utilizado prostaglandinas con fines de acelerar el movimiento ortodóntico en humanos, comenzando con Yamasaki *et al*. ^10^, quienes administraron 10 *u*g de PGE1 en 9 pacientes con extracción de primeros premolares, que contaban con aparatología fija, un arco transpalatino y un arco continuo. Un resorte de níquel titanio abierto fue introducido para distalizar caninos y ejerció una fuerza de 150 g. Al cabo de 3 meses y medio, se reportó una media de movimiento dentario para PGE1 de 2,07 mm y 1,30 mm para el lado control. No se reportó irritación de los tejidos blandos.

Spielmann *et al*. ^31^ administraron 10 *u*g de PGE1 semanalmente a 5 pacientes, a los que se les programó las extracciones de primeros premolares superiores como parte del tratamiento ortodóntico. Se instalaron aparatos (botones adhesivos) en la superficie lingual de los primeros premolares y se generó una tracción en sentido transversal hacia medial, mediante una cadena elástica que fue cambiada semanalmente. Las dosis fueron inyectadas en la mucosa palatal de un lado y el lado contralateral fue el control. El movimiento dental registrado fue de 3,01 mm (PGE1) y el lado control fue de 1,03 mm. No se registraron efectos negativos en los tejidos ni alteraciones radiológicas.

Patil *et al.*^11^ evaluaron los efectos de la PGE1 en 14 pacientes a los cuales se les extrajo los primeros premolares como parte del tratamiento ortodóntico. Se administró 3 dosis de 1 *u*g en la mucosa del vestíbulo. Los pacientes tenían aparatología fija, un arco transpalatino y se ligaron las segundas molares para incrementar el anclaje. Se realizó la tracción de los caninos por medio de un resorte de níquel titanio cerrado (150 g) y, al cabo de 60 días, se realizaron las medidas comparativas. Se encontró que el desplazamiento dentario para la PGE1 fue de 3,5 mm, mientras que para el lado control fue de 2 mm. No se evidenció reabsorción radicular en radiografías periapicales.

Rajasekaran *et al*. ^32^ compararon el movimiento dentario (retracción de caninos) en 32 pacientes a los que se les administró, por vía submucosal, una dosis de 100 *u*g de PGE1 en el lado derecho y se realizó una corticotomía de colgajo completo en el lado izquierdo. Los pacientes contaban con aparatología fija, un arco de acero .017 x .025 y un resorte cerrado de níquel titanio (100 g). Las dosis de la PGE1 fueron administradas cada 2 semanas, se tomaron modelos de estudio semanalmente y radiografías periapicales al final de la etapa de cierre de espacios. Se reportó un movimiento dentario para la corticotomía (0,4 mm ± 0,04 por semana) y para la PGE1 (0,36 mm ± 0,05 por semana), así como la cantidad de reabsorción radicular para la corticotomía (0,4 mm) y para la PGE1 (0,5 mm). 

Jain *et al*. ^33^ administraron 3 *u*g de PGE1 en formulación tipo gel a 15 pacientes con aparatología fija en fase de retracción de caninos. Los pacientes contaban con un arco de acero .019 x .025 y un arco transpalatino. La aplicación de PGE1 se realizó de manera tópica cada 15 días sobre la zona a evaluar, siendo la hemiarcada opuesta el lado control. Al cabo de 2 meses, se obtuvo un desplazamiento del canino en el lado experimental de 2,34 mm, y en el lado control, de 1,7 mm. Los pacientes no experimentaron dolor ni efectos secundarios evidentes.

## DISCUSIÓN

El propósito del presente estudio es brindar información actualizada acerca del uso de las prostaglandinas, su mecanismo de acción, sus efectos en el movimiento dentario y su aplicación clínica, para lo que se realizó una revisión exhaustiva de la literatura. Sin embargo, la cantidad de artículos relacionados con el tema fue reducida y no se encontraron estudios de largo plazo. 

Los artículos analizados en la presente revisión de la literatura reportaron que hay una relación directa entre la aplicación de prostaglandinas y un aumento en la velocidad del desplazamiento dentario, en comparación con el grupo control (que contaba con aparatología ortodóntica sin administración de algún compuesto farmacológico). Se reportó que el número de osteoclastos aumentó en la zona de compresión por acción de las prostaglandinas, la cual ejerce un efecto vasodilatador, lo que incrementa el aporte sanguíneo y fomenta la presencia de agentes inflamatorios ^34^. Esto produce una tasa acelerada de reabsorción ósea y un desplazamiento dentario que, según la literatura, es entre 1,5 a 3 veces más veloz que la velocidad normal ^10^^,^^31^.

En relación con la metodología utilizada en la experimentación con animales (en su mayoría ratas), algunos estudios no mencionan la edad de los animales, un factor de gran importancia al momento de evaluar la actividad metabólica durante la remodelación ósea, siendo los animales jóvenes los que muestran una mayor actividad. Adicionalmente, las fuerzas ortodónticas empleadas en los experimentos en animales sobrepasan las consideradas ideales para roedores. Según la literatura, la fuerza necesaria para desplazar dientes en ratas es de 10 a 20 g ^12^, mientras que, en los estudios experimentales con prostaglandinas, las fuerzas aplicadas oscilaron entre los 50 a 80 g. Esto podría explicar la evidencia de reabsorción radicular en los grupos experimentales ^35^^,^^36^. 

Con respecto a las vías de administración en los estudios experimentales, la vía más común fue la inoculación submucosal; sin embargo, se ha reportado que la vía de administración local más eficaz es la intraligamental, por presentar un incremento en el número de osteoblastos y osteoclastos ^21^. La administración de PGE1 en su presentación tipo gel presenta una biodisponibilidad menor; sin embargo, no presenta los efectos indeseados como el dolor ni la necesidad de inocular al paciente ^33^. La administración por vía oral del compuesto análogo de la PGE1, misoprostol, no ha reportado evidencia de reabsorción radicular, si bien solo se cuenta con un estudio en animales ^29^.

En los estudios realizados en humanos se utilizaron diferentes dosis, desde 1 *u*g a 100 *u*g, por la vía submucosal y tópica, y se reportó un aumento en el movimiento dentario en todos los casos; no obstante, es necesario determinar las dosis adecuadas y realizar estudios longitudinales para determinar su efectividad como coadyuvante de las mecánicas ortodónticas ^37^. La combinación de prostaglandinas con gluconato de calcio podría presentarse como una alternativa más segura frente al riesgo de reabsorción radicular ^22^^,^^25^.

La evaluación de la reabsorción radicular requiere examinar la anatomía radicular en 3 dimensiones ^34^, y resulta insuficiente evaluar imágenes bidimensionales como las radiografías periapicales (las cuales presentan magnificaciones, deformaciones y problemas de posicionamiento) para determinar con certeza la presencia y magnitud de la reabsorción radicular, la cual puede ser evidente radiográficamente una vez perdido un porcentaje significativo del tejido radicular ^38^^-^^40^. 

Se hace evidente la necesidad de emplear métodos más sensibles para la identificación de la reabsorción radicular y que, a su vez, no generen una exposición excesiva a la radicación ionizante, por lo que la tomografía *cone beam* no parece presentarse como una alternativa viable para la detección temprana de reabsorción radicular, debido a su alto costo económico y radicación. Sin embargo, en presencia de una reabsorción radicular activa, las imágenes tomográficas pueden ayudar a determinar el porcentaje de la superficie radicular que se encuentra afectada. Algunos autores han propuesto la posibilidad de utilizar métodos menos invasivos para la detección de reabsorción radicular, como es el estudio de los biomarcadores del fluido crevicular gingival, puesto que son de simple ejecución y fáciles de reproducir ^40^^-^^42^.

Las revisiones sistemáticas respecto al tema de investigación han encontrado limitaciones al momento de realizar conclusiones, debido al riesgo de sesgo, la falta de estandarización y la limitada muestra, siendo esta una de las limitaciones del presente estudio ^43^.

### Limitaciones

La presente revisión de la literatura tuvo como limitación el escaso número de artículos referentes al tema de investigación. Las experimentaciones en humanos son escasas y han tenido una muestra pequeña, lo que genera dificultades para obtener resultados. 

## CONCLUSIONES

La administración local de prostaglandinas por la vía submucosal ha demostrado acelerar el movimiento dentario. No se han reportado lesiones en los tejidos de soporte dentario después de la administración local de prostaglandinas en humanos; sin embargo, la inoculación de dosis altas ha mostrado evidencia de reabsorción radicular en animales. La combinación de prostaglandinas y gluconato de calcio ha demostrado reducir la reabsorción radicular mientras aumenta la velocidad del movimiento dentario en animales. Dosis únicas de prostaglandinas han mostrado un efecto similar al de dosis repetitivas. 

## Financiamiento:

Autofinanciado
